# The Challenge of Converting “Failed Spinal Cord Stimulation Syndrome” Back to Clinical Success, Using SCS Reprogramming as Salvage Therapy, through Neurostimulation Adapters Combined with 3D-Computerized Pain Mapping Assessment: A Real Life Retrospective Study

**DOI:** 10.3390/jcm11010272

**Published:** 2022-01-05

**Authors:** Philippe Rigoard, Amine Ounajim, Lisa Goudman, Tania Banor, France Héroux, Manuel Roulaud, Etienne Babin, Bénédicte Bouche, Philippe Page, Bertille Lorgeoux, Sandrine Baron, Nihel Adjali, Kevin Nivole, Mathilde Many, Elodie Charrier, Delphine Rannou, Laure Poupin, Chantal Wood, Romain David, Maarten Moens, Maxime Billot

**Affiliations:** 1 PRISMATICS Lab (Predictive Research in Spine/Neuromodulation Management and Thoracic Innovation/Cardiac Surgery), Poitiers University Hospital, 86021 Poitiers, France; amine.ounajim@chu-poitiers.fr (A.O.); Manuel.ROULAUD@chu-poitiers.fr (M.R.); etienne.babin82@gmail.com (E.B.); dr.bouche@gmail.com (B.B.); Bertille.LORGEOUX@chu-poitiers.fr (B.L.); sandrine.baron@chu-poitiers.fr (S.B.); Nihel.adjali@chu-poitiers.fr (N.A.); Kevin.NIVOLE@chu-poitiers.fr (K.N.); mathilde.many@chu-poitiers.fr (M.M.); chantalwood@orange.fr (C.W.); romain-david@hotmail.fr (R.D.); 2 Department of Spine Surgery & Neuromodulation, Poitiers University Hospital, 86021 Poitiers, France; tania.banor@gmail.com (T.B.); Philippe.PAGE@chu-poitiers.fr (P.P.); 3 Pprime Institute UPR 3346, CNRS, ISAE-ENSMA, University of Poitiers, 86360 Chasseneuil-du-Poitou, France; 4 Laboratoire de Mathématiques et Applications, UMR 7348, Poitiers University and CNRS, 86000 Poitiers, France; 5 Department of Neurosurgery, Universitair Ziekenhuis Brussel, 1090 Brussels, Belgium; lisa.goudman@gmail.com (L.G.); mtmoens@gmail.com (M.M.); 6 STIMULUS Research Group, Vrije Universiteit Brussel, 1090 Brussels, Belgium; 7 Department of Neurosurgery, Sherbrooke University, Saguenay Delocalized Site, Chicoutimi Hospital, Sherbrooke, QC G7H 5H6, Canada; france.heroux@usherbrooke.ca; 8 Pain Evaluation and Treatment Centre, Poitiers University Hospital, 86021 Poitiers, France; Elodie.CHARRIER@chu-poitiers.fr (E.C.); delphine.rannou@chu-poitiers.fr (D.R.); laure.poupin@chu-poitiers.fr (L.P.); 9 Physical and Rehabilitation Medicine Unit, Poitiers University Hospital, University of Poitiers, 86021 Poitiers, France

**Keywords:** rescue therapy, paresthesia-free waveforms, high-frequency, burst, spatial neural targeting, temporal neural targeting, SCS programming, mapping software, paresthesia coverage, salvage algorithm

## Abstract

While paresthesia-based Spinal Cord Stimulation (SCS) has been proven effective as treatment for chronic neuropathic pain, its initial benefits may lead to the development of “Failed SCS Syndrome’ (FSCSS) defined as decrease over time related to Loss of Efficacy (LoE) with or without Loss of Coverage (LoC). Development of technologies associating new paresthesia-free stimulation waveforms and implanted pulse generator adapters provide opportunities to manage patients with LoE. The main goal of our study was to investigate salvage procedures, through neurostimulation adapters, in patients already implanted with SCS and experiencing LoE. We retrospectively analyzed a cohort of patients who were offered new SCS programs/waveforms through an implanted adapter between 2018 and 2021. Patients were evaluated before and at 1-, 3-, 6- and 12-month follow-ups. Outcomes included pain intensity rating with a Visual Analog Scale (VAS), pain/coverage mappings and stimulation preferences. Last follow-up evaluations (N = 27) showed significant improvement in VAS (*p* = 0.0001), ODI (*p* = 0.021) and quality of life (*p* = 0.023). In the 11/27 patients with LoC, SCS efficacy on pain intensity (36.89%) was accompanied via paresthesia coverage recovery (55.57%) and pain surface decrease (47.01%). At 12-month follow-up, 81.3% preferred to keep tonic stimulation in their waveform portfolio. SCS conversion using adapters appears promising as a salvage solution, with an emphasis on paresthesia recapturing enabled via spatial retargeting. In light of these results, adapters could be integrated in SCS rescue algorithms or should be considered in SCS rescue.

## 1. Introduction

Paresthesia-based Spinal Cord Stimulation (SCS) is a well-established therapy with robust clinical evidence aimed at treating patients suffering from refractory Failed Back Surgery Syndrome(FBSS)/Persistent Spinal Pain Syndrome after surgery (PSPS-T2) [1,2,3,4]. In some cases, the initial benefits of the therapy may decrease over time, described as “Failed SCS Syndrome” (FSCSS), which may lead to explantation of the device [5,6,7]. In a retrospective review reporting long-term outcomes in 955 SCS patients, Van Buyten et al. [8] documented and analyzed the reasons for which a small but substantial proportion of patients have been explanted over time (7.9% per year). Besides the explants related to complications (hardware dysfunction, cerebrospinal fluid leak, documented allergy to device components or infection), this study group also reported that over half of explants (94/180) were performed because patients had lost the analgesic benefits of SCS therapy over time, described as Loss of Efficacy (LoE).

LoE may happen over time for various reasons [9,10] and can be dichotomized into two different subcategories of patients. For patients using paresthesia-based stimulation, therapy benefits may be reduced over time due to a Loss of pain Coverage (LoC) [10,11,12]. Some patients may experience a new onset of pain or a change in their initial pain pattern. For other patients belonging to this subgroup, despite a stable pain condition, they can no longer perceive their stimulation (paresthesia) in residual painful territories, which are adequately covered by SCS initially. For these scenarios, when reprogramming remains unsuccessful in “recapturing” the residual painful area, this Lack of Coverage (LoC) of optimal paresthesia usually leads to LoE. A second category includes patients who, contrary to the first group, do not experience satisfactory pain relief and are no longer responsive to SCS therapy (LoE patients), despite adequate pain coverage (no LoC). In the long term, these patients may decrease their use of stimulation or abandon the therapy. We could describe this group as patients experiencing a real “tolerance” to the treatment [13,14,15], i.e., to the electrical delivery of SCS.

Currently, EC-marked (European Conformity) adapters can be used for patients implanted with an SCS device, enabling clinicians to upgrade the Internal Pulse Generator (IPG) with a different stimulation technology, without changing the existing lead(s). With a simple surgical procedure, previously inaccessible SCS programs and waveforms can be used to regain pain relief [16,17]. Proposing new SCS waveforms to rescue patients presenting with FSCSS was recently introduced in clinical practice and showed promising results and significant improvements in patient outcomes [6,11,18,19,20]. However, the dichotomy between the two above-mentioned sub-categories of FSCSS patients, who might both benefit from this new strategy (i.e., LoE with LoC or LoE without LoC), has never been precisely examined as regards the need for spatial retargeting (using electrical field fragmentation) and/or temporal adjustments (using new waveforms).

We designed a retrospective study to further investigate the potential benefit of new spatial and temporal SCS paradigms proposed to patients for whom SCS has shown LoE. Patients previously implanted with an SCS system, which no longer provides adequate pain relief (global VAS > 4), were implanted and assessed with a 12-month follow-up period to confirm and to further characterize the outcomes of therapy conversions. In addition, our team created a 3D-computerized triple-patented pain mapping tool [21], to be able to document precise paresthesia coverage in cm², pain surface and pain typology changes over time, using objective quantitative metrics, via a tactile interface. These quantitative measurements can be correlated to classical pain assessment tools, such as pain intensity, functional incapacity, quality of life and patient satisfaction, to build a multi-dimensional composite index [22], aimed at assessing pain, therapy efficacy and patient outcomes in a holistic manner. Finally, a new proposal for a salvage algorithm was presented and will be discussed, hereafter, as a synthesis of the discussion.

## 2. Material and Methods

### 2.1. Study Design

A retrospective monocentric, observational, longitudinal, real-life cohort study including all consecutive patients, in whom an adapter had been implanted in our department (Poitiers University Hospital, France) was conducted between 2018 and 2021. Written informed consent was obtained from all patients included in the study before data collection. The study was conducted in accordance with the Declaration of Helsinki, and the protocol was approved by the Ethics Committee of the Poitiers University Hospital (N° F20210507150101). The study did not receive any financial support from industry and industry did not participate in data collection or in data analysis.

### 2.2. Study Population

Patients meeting the following criteria were included in the study: presents refractory neuropathic chronic pain (i.e., regardless of the localization of the pain); had been implanted with a previous SCS system and had demonstrated significant pain relief following their lead trial and remained postoperatively satisfied with the stimulation for months or years; had experienced LoE over time and were finally considered as FSCSS patients, despite SCS initial success; evaluated by a multidisciplinary team in order to exclude psychological (e.g., new traumatic event) and financial (e.g., secondary gain) reasons that might explain SCS loss of efficacy and to rule out hardware dysfunctions; were eligible for an SCS adapter implantation.

Two groups of patients were identified:

− The “loss of coverage” group (LoC group) including patients for whom pain coverage was not adequate (<60%).
− The “SCS tolerance” group (SCStol group), including patients with adequate pain coverage for whom SCS no longer provided pain relief.

### 2.3. Clinical Strategy and Procedures

Prior to considering an adapter implantation, patients previously implanted with an SCS device and still presenting with refractory chronic neuropathic pain due to LoE had to be reassessed in a multidisciplinary pain clinic to (i) identify the potential cause of LoE, (ii) rule out any hardware dysfunction and (iii) investigate any reprogramming issues or lead impedance. Furthermore, the absence of lead displacement was systematically assessed using recent imaging data, especially in the case of loss of coverage. In the case of a negative issue managing persistent pain, patients were proposed to be implanted with an adapter (Boston Scientific VerciseTM M8 Adapter or PrecisionTM S8 adapter). An externalized temporary generator was implanted for a seven-day SCS screening trial period. The adapter screening test was considered as positive if the patient achieved a global pain VAS score decrease ≥50% after a seven-day period trial according to the French Health Authority guidelines or if the improvement was clinically important according to patient goals and expectations and was permanently implanted with a new SCS device at the end of the SCS adapter screening trial. Patients who did not achieve 50% pain reduction or clinically important improvement were proposed to either get their previous SCS device reconnected or completely explanted (lead(s) was either explanted or not, based on patient preference).

All patients receiving an adapter IPG were upgraded with the same technology (Spectra Wavewriter, Boston Scientific, Malborough, MA, USA) enabling spatial and temporal re-targeting, using various reprogramming strategies: the shape of the electrical field could be adapted using Multiple Independent Current Control (MICCTM) technology to optimize spatial targeting [17], and one or several waveform(s) and combinations of tonic, burst or HF waveforms were proposed to the patient [16], to adjust temporal resolution of the signal.

Patients were assessed at baseline (before adapter implantation), and at 1, 3, 6 and 12 months of follow-up. During these visits, patients reported the efficacy of their stimulation and change waveforms if needed. During the programming sessions of the visits, new waveforms, combined or not, were randomly proposed to the patient. The patient reported verbally if the stimulation was able to cover his/her painful area and finally chose the program he/she preferred. Programming sessions lasted about 45 min.

### 2.4. Outcome Measurements

**Figure 1:**
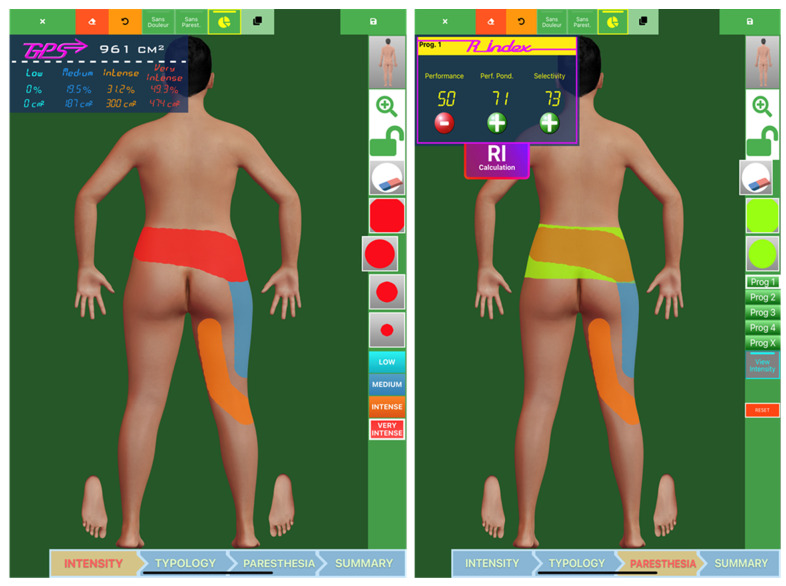
Pain mapping software used to assess pain surface and pain coverage where the patient could draw different painful zones. The pixels in the patient drawing are then converted into cm², using several anatomical landmarks, patient morphology and morphometry. Four colors are available for patients to represent the different pain intensities. Pain coverage can then be obtained by drawing paresthesia (in green), which is converted to a percentage of pain coverage (performance).

Pain intensity was assessed using a global Visual Analogic Scale (VAS), functional disability using the Oswestry Disability Index (ODI), health-related quality of life using the EuroQuol-5 Dimensions 5 Levels (EQ-5D-5L) questionnaire, depression and anxiety levels using the Hospital Anxiety and Depression Scale (HADS), and patient satisfaction using the Patient Global Impression of Change (PGIC), which measures the patient’s belief about the efficacy of treatment (ranging from 1 “No improvement at all or even worse” to 7 “Very important improvement which makes all the difference”). Pain surface area and paresthesia coverage were collected from a touch screen, where the patient drew the various pain and paresthesia areas, which were then represented in maps and diagrams. The pixels of the patient’s drawing were then converted to cm², using several anatomical landmarks, patient morphology and morphometry, to optimally and accurately measure the pain area, using a patented data processing system (Patent Applications N° PCT/EP2014/067231, N° PCT/FR 14/000 186 and N° PCT/FR 14/000 187) [21]. The pain mapping software provides objective and reproducible measurements about the total pain surface and pain surface related to pain intensity expressed in cm², paresthesia coverage as the percentage of pain surface covered by paresthesia and the typology of pain (Figure 1) [21,22,23,24]. Paresthesia coverage was considered as adequate when the paresthesia performance was greater than 60% (i.e., when at least 60% of the pain surface was covered with paresthesia). When data were missing, the patient was contacted by a clinical research associate in order to complete the data collection. When the patient was reachable, their data was collected over the phone by a clinical research associate who completed the series of questionnaires and pain mappings, using our touchscreen computer interface. When we could not reach the patient, the patient was considered as lost to follow-up.

### 2.5. Statistical Analysis

R software (Version 3.6.1, R Foundation for Statistical Computing, Vienna, Austria) was used for the statistical analysis.

Quantitative variables were described using mean and its standard deviation (SD) and qualitative variables were described using the number of patients and their percentage in each modality.

The significance of global change of each of the study outcomes (VAS, ODI, EQ-5D, HADS and pain surface) following implantation of an adapter was evaluated by applying a mixed effect regression model with the follow-up visit (all visits analyzed simultaneously) as a fixed effect variable and a random patient-specific intercept to account for within-patient dependencies. When the follow-up effect was significant, each follow-up visit was compared to baseline using a paired *t*-test or a Wilcoxon signed-rank test, depending on the normality of distributions. Normality of distributions was tested using the Shapiro–Wilk test. The confounding factors age, sex and time between the previous SCS system implantation and the adapter implantation were tested and were not statistically clinically significant. For this reason, and to improve interpretability, we have chosen not to include the confounder factors in this study to improve interpretability [25].

Three approaches were used to treat missing data. The first analysis consisted of using only available cases for each follow-up. This approach might lead to biased results (i.e., non-responders usually stop attending their follow-up visits). Therefore, a second analysis was carried out using data from the last available observation of the patient for the remaining follow-up visits (last observation carried forward method). The seven-day screening trial outcome was considered as the last observation for patients with a negative screening trial. The third approach is a worst-case analysis [26,27]. In this analysis, data for patients with missing follow-up were imputed using the baseline observation prior to the adapter implantation (i.e., we assume that data are missing because the patient is no longer satisfied with his/her new stimulation device).

We also compared baseline characteristics, percentage of pain surface decrease, paresthesia coverage and percentage of pain intensity decrease between the LoC group and the SCStol group for each follow-up visit (at 1, 3, 6 and 12 months). For quantitative variables, these comparisons were conducted using a *t*-test or a Mann–Whitney test depending on the normality of distributions. An exact Fisher test was used to compare qualitative variables between the two groups.

All tests were conducted as two-tailed. A *p*-value of less than 0.05 was considered as statistically significant. As recommended by Rothman et al. [28] and Althouse [29], no *p*-value adjustment for multiple comparisons was considered in this exploratory study with preplanned outcomes and analysis.

## 3. Results

### 3.1. Baseline Characteristics

**Figure 2:**
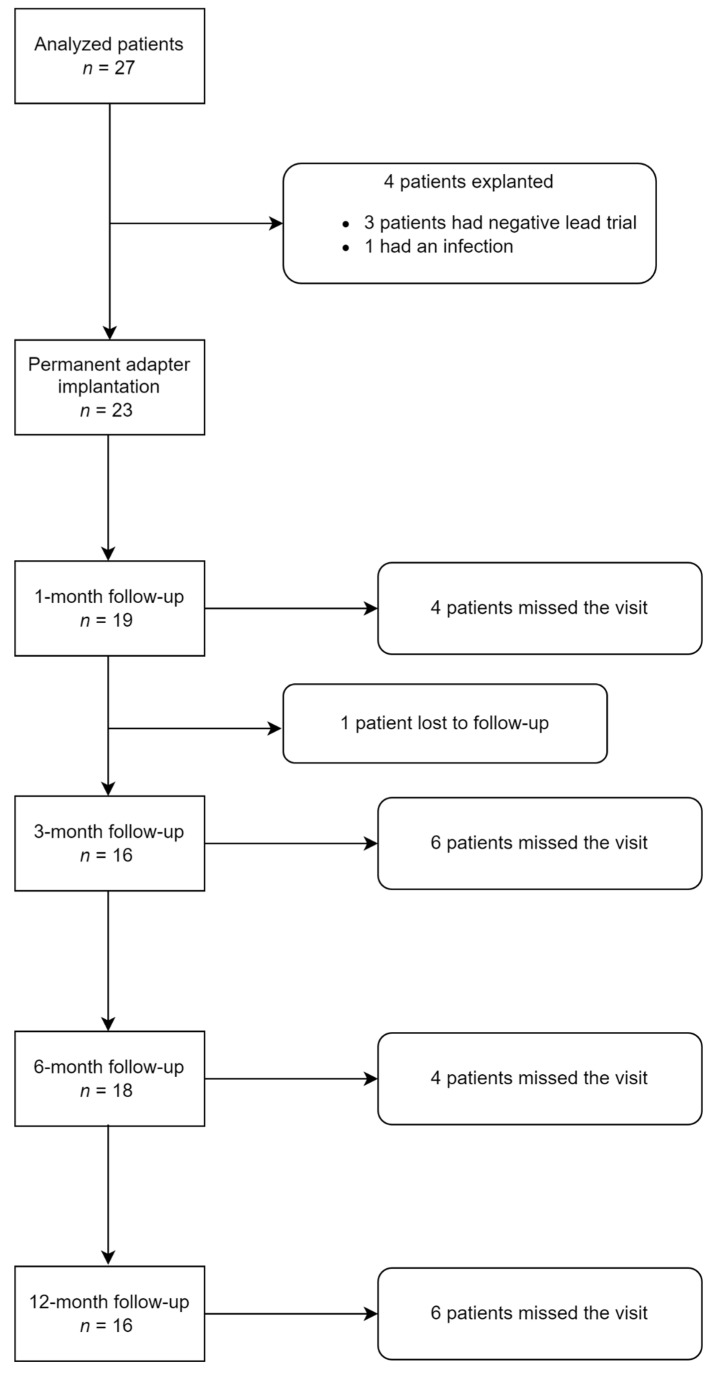
Patient flow chart.

Baseline characteristics of the patients and the study follow-up are presented in Table 1 and Figure 2, respectively. Twenty-seven patients having undergone an adapter implantation in Poitiers University Hospital were identified during the inclusion period and provided their consent to participate. The screening trial phase was successful in 23/27 patients (85.2%), implanted with a new stimulator: 3 patients (11.1%) did not achieve adequate pain relief following a screening trial and 1 patient (3.7%) was explanted due to infection following lead externalization. Among the 23 patients who received a permanent IPG, 19 patients (82.6%) were assessed at 1-month, 16 patients (69.6%) at 3-month, 18 patients (78.3%) at 6-month and 16 patients (69.6%) at 12-month follow-up (the restrictive displacement during the pandemic explains the high rate of data loss). The mean age of the 27 analyzed patients was 53.2 ± 11.6 years, and 15/27 (55.6%) of our patients were male. Nineteen patients (70.4%) had back and leg pain, 2 (7.4%) had only leg pain, 3 patients (11.1%) had upper limb neuropathic pain, 2 patients (7.4%) had groin pain and 1 patient (3.7%) had a cluster headache. The mean duration between the initial SCS implantation and the adapter implantation was 5.9 ± 5.2 years. Twenty-six patients used tonic conventional stimulation before adapter implantation, while 1 patient used tonic and HD stimulations waveforms. Sixteen out of 27 (59.3%) patients had adequate pain coverage (>60%) and were allocated to the SCStol group. The 11 remaining patients were allocated to the LoC group. Before adapter implantation, mean performance of the initial paresthesia coverage (percentage of pain surface covered by SCS paresthesia) was 37.8 ± 39.5%.

### 3.2. Pain Related Outcome following Device Adapter

#### 3.2.1. Available Case Analysis

Results of paired comparisons for 1-month, 3-month, 6-month and 12-month follow-ups are presented in Table 2 for all outcomes.

**Figure 3:**
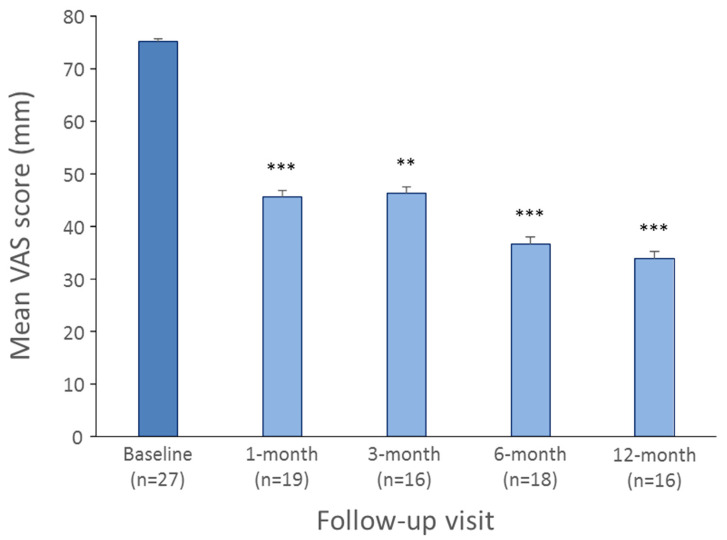
Mean VAS scores (and its standard error) obtained at baseline and at each follow-up visit. *** *p* < 0.001, ** *p* < 0.01 significant difference between baseline and follow-up visits.

For the global VAS, the mixed effects model showed a significant time effect (*p* < 0.0001). The VAS significantly decreased from baseline to 1-month (*n* = 19, 75.26 ± 16.45 mm vs. 45.56 ± 24.31 mm, *p* = 0.001), 3-month (*n* = 16, 71.88 ± 16.82 mm vs. 46.25 ± 19.96 mm, *p* = 0.0045), 6-month (*n* = 18, 78.33 ± 12.0 mm vs. 36.67 ± 23.26 mm, *p* < 0.0001) and 12-month follow-up (*n* = 16, 78.12 ± 11.09 mm vs. 33.75 ± 21.87, *p* < 0.0001) (Figure 3).

For the ODI score, the mixed effects model showed a significant time effect (*p* = 0.0001). A significant decrease in ODI was also observed from baseline to 1-month (*n* = 19, 48.81 ± 12.55 vs. 31.69 ± 12.78; *p* = 0.0014), 3-month (*n* = 16, 45.4 ± 16.97 vs. 28.12 ± 12.36; *p* = 0.0195) and 6-month follow-up (*n* = 18, 43.11 ± 15.77 vs. 31.9 ± 13.85, *p* = 0.0423). The decrease did not persist at 12-month follow-up (*n* = 16, 47.71 ± 14.62 vs. 34.0 ± 22.84, *p* = 0.098).

For the EQ5D index, the mixed effects model showed a significant time effect (*p* < 0.0001). The EQ-5D index increased significantly from baseline to 1-month (*n* = 19, 0.25 ± 0.17 vs. 0.53 ± 0.23; *p* = 0.0038), 3-month (*n* = 16, 0.29 ± 0.21 vs. 0.53 ± 0.19; *p* = 0.0207) and 6-month (*n* = 18, 0.32 ± 0.22 vs. 0.53 ± 0.26; *p* = 0.0223) but not at 12-month follow-up (*n* = 16, 0.27 ± 0.19 vs. 0.48 ± 0.36; *p* = 0.18).

For the HADS depression score, the mixed effects model showed a significant time effect (*p* = 0.012). The HADS depression score also improved significantly from baseline to 1-month (*n* = 19, 5.82 ± 3.52 vs. 3.6 ± 2.56, *p* = 0.0442), 3-month (*n* = 16, 6.08 ± 3.87 vs. 3.5 ± 3.03, *p* = 0.0421) and 6-month follow-up (*n* = 18, 5.07 ± 3.34 vs. 4.3 ± 3.89, *p* = 0.0192) but not 12-month follow-up (*n* = 16, 6.33 ± 3.85 vs. 5.0 ± 5.55, *p* = 0.13).

For the HADS anxiety score, the mixed effects model showed a significant time effect (*p* = 0.002). The HADS anxiety score improved significantly from baseline to 1-month follow-up (*n* = 19, 8.88 ± 4.55 vs. 6.53 ± 3.78, *p* = 0.0488) but not at 3-month (*n* = 16, 8.0 ± 4.84 vs. 6.7 ± 3.8, *p* = 0.0562), 6-month (*n* = 18, 7.93 ± 4.62 vs. 6.6 ± 6.15, *p* = 0.0545) and 12-month follow-up (*n* = 16, 8.75 ± 5.19 vs. 8.0 ± 5.8, *p* = 0.18).

For the total pain surface, the mixed effects model showed a significant time effect (*p* = 0.006). The total pain surface significantly decreased from baseline to 1-month (*n* = 19, 938.26 ± 840.56 cm² vs. 441.82 ± 597.72 cm², *p* = 0.0013), 3-month (*n* = 16, 919.75 ± 926.34 cm² vs. 569.79 ± 878.56 cm², *p* = 0.0144) and 12-month follow-up (*n* = 16, 887 ± 804.4 cm² vs. 545.12 ± 859.58 cm², *p* = 0.0115), but not 6-month follow-up (*n* = 18, 848.22 ± 778.08 cm² vs. 711.89 ± 969.23 cm², *p* = 0.0883).

For the “very intense” pain surface, the mixed effects model showed a significant time effect (*p* = 0.003). The very intense pain surface significantly decreased from baseline to 1-month (*n* = 19, 638.33 ± 862.57 cm² vs. 28.24 ± 82.65 cm², *p* = 0.0021), 3-month (*n* = 16, 493.12 ± 920.89 cm² vs. 288.5 ± 913.49 cm², *p* = 0.021), 6-month (*n* = 18, 562.53 ± 903.3 cm² vs. 223.61 ± 809.48 cm², *p* = 0.021) and 12-month follow-up (*n* = 16, 573.4 ± 950.46 cm² vs. 240.12 ± 858.75 cm², *p* = 0.0191).

#### 3.2.2. Last Observation Carried Forward Analysis

In this analysis, we considered the last follow-up available for each patient. Seven-day screening trial data was considered as the last follow-up for patients who had a negative screening trial (*n* = 3), were explanted due to an infection (*n* = 1) and patients for whom no follow-up was available (*n* = 2).

Differences for global VAS, ODI score, EQ-5D index, HADS depression and anxiety scores and pain surface between baseline and the last follow-up are presented in Table 3. We observed a significant improvement from baseline to the last follow-up for the global VAS (75.1 ± 14.9 mm vs. 39.1 ± 27.9 mm, *p* = 0.0001), the ODI score (48.5 ± 15.9% vs. 38.9 ± 20.5%, *p* = 0.021), the EQ-5D-5L index (0.25 ± 0.2 vs. 0.39 ± 0.3, *p* = 0.023), the HADS anxiety score (8.52 ± 4.32 at baseline to 7.5 ± 4.15, *p* = 0.036) and the pain surface (1085.19 ± 1360.69 cm² vs. 718.85 ± 1430.0 cm², *p* = 0.0013). However, the HADS depression score did not change significantly between baseline and the last follow-up (6.19 ± 3.71 vs. 5.08 ± 4.55, *p* =0.067).

#### 3.2.3. Worst-Case Analysis

In this analysis, patients with missing data were considered as non-responders and baseline data were used to impute the missing follow-up data for each patient.

Worst-case analysis showed a significant improvement of the global VAS from baseline (75.1 ± 14.9 mm) to 1 month (55.1 ± 24.7 mm, *p* = 0.0011), 3 months (59.9 ± 23.5 mm, *p* = 0.0045), 6 months (47.3 ± 26.3 mm, *p* = 0.0006) and 12 months (48.8 ± 27.4 mm at, *p* = 0.0007). The ODI score significantly improved from baseline (48.5 ± 15.9%) to 1 month (37.4 ± 17.8% at 1-month, *p* = 0.001), 3 months (38.1 ± 17.9%, *p* = 0.024) and 6 months (43.5 ± 17.3%, *p* = 0.042), but did not significantly differ after 12 months (43.6 ± 18.2%, *p* = 0.098). The EQ-5D-5L index significantly improved from baseline (0.25 ± 0.2) to 1 month (0.44 ± 0.27, *p* = 0.004), 3 months (0.38 ± 0.24, *p* = 0.021) and 6 months (0.34 ± 0.27, *p* = 0.022), but was not significantly different after 12 months (0.30 ± 0.25, *p* = 0.18). The HADS depression score significantly improved from baseline (6.2 ± 3.7) to 1 month (04.8 ± 3.4, *p* = 0.044), 3 months (4.9 ± 3.6, *p* = 0.042) and 6 months (5.9 ± 4.0, *p* = 0.019), but was not significantly different after 12 months (5.5 ± 3.9, *p* = 0.10). The pain surface significantly decreased from baseline (1085.2 ± 1360.7 cm²) to 1 month (743.1 ± 1318.5 cm², *p* = 0.0029), 3 months (962.9 ± 1387.8 cm², *p* = 0.014) and 12 months (882.6 ± 1420.3 cm², *p* = 0.011), but was not significantly different after 6 months (994.3 ± 1455.6 cm², *p* = 0.088).

### 3.3. Comparisons between the “Loss of Coverage” Group and the “SCS Tolerance” Group

#### 3.3.1. Outcome Comparison

In this analysis, we compared the pain relief, pain surface decrease and paresthesia coverage between the LoC group (*n* = 11) and the SCStol group (*n* = 16). Table 4 presents the results of the comparisons between the two groups. We found significant differences between the groups in the percentage of pain surface decrease at 1 month (64.3 ± 33.9 cm² in the LoC group vs. 11.77 ± 65.8 cm² in the SCStol group, *p* = 0.034) and at 3 months (47.01 ± 38.69 in the LoC group against 20.13 ± 69.85 in the SCStol group, *p* = 0.047). The patients in the LoC group had significantly better paresthesia coverage at 1-month follow-up than the SCStol group (*p* = 0.048). We did not find any other significant difference in the analysis in the percentage of VAS decrease at all follow-ups and for the percentage of pain surface and paresthesia coverage at 6- and 12-month follow-ups.

#### 3.3.2. Waveform Comparison

**Figure 4:**
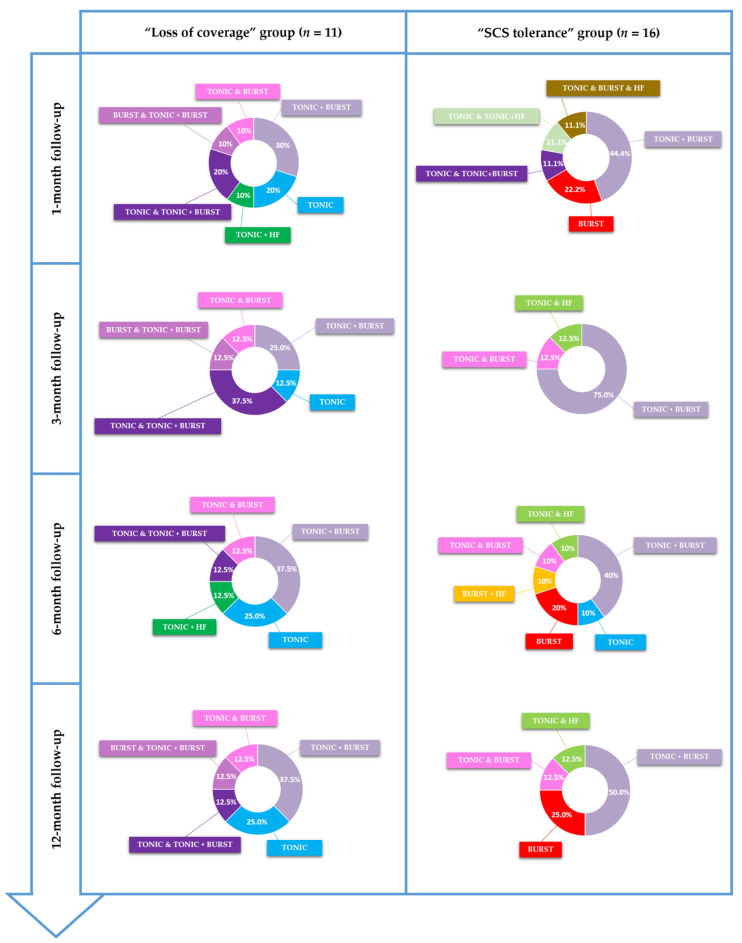
Pie chart of the waveforms used by the “loss of coverage” and “SCS tolerance” groups for each follow-up. The sign “+” represents combination therapies (i.e., when two waveforms are used simultaneously) and the sign “&” represents programs that are used separately (e.g., TONIC & HF might be TONIC during the day and HF during night).

The comparisons between the two groups of waveforms applied can be found in Figure 4. At the 1-month follow-up, in the LoC group (*n* = 10), 6 patients (60%) used only one program while 4 patients (40%) preferred to have 2 programs in their stimulation portfolio. Three patients (30%) used only TONIC + BURST simultaneously. TONIC stimulation only was used by 2 patients (20%). TONIC + HF simultaneous combination was used by 1 patient (10%). Among the 4 patients who used 2 programs, 2 patients (20%) used TONIC + BURST combination and TONIC, 1 patient (10%) used TONIC + BURST combination and BURST and 1 patient (10%) used TONIC and BURST. In the SCStol group (*n* = 9), 6 patients (66.7%) used only one program while 2 patients (22.2%) used 2 programs and 1 patient (11.1%) used 3 programs. Four patients (44.4%) used only the TONIC + BURST combination therapy and 2 patients (22.2%) used only BURST stimulation. Among the 2 patients who preferred to have 2 programs in their stimulation portfolio, 1 patient (11.1%) used TONIC stimulation and TONIC + HF combination therapy and 1 patient (11.1%) used TONIC and TONIC + BURST combination. The 1 patient (11.1%) who preferred to use 3 programs used TONIC, BURST and HF stimulation.

At the 3-month follow-up, in the LoC group (*n* = 8), 3 patients (37.5%) used only one program while 5 patients (62.5%) preferred to have 2 programs in their stimulation portfolio. Two patients (25%) used only TONIC + BURST simultaneously and 1 patient (12.5%) used TONIC stimulation only (below the paresthesia threshold). Among the 5 patients who used 2 programs, 3 patients (37.5%) used TONIC + BURST combination and TONIC stimulation, 1 patient (12.5%) used TONIC + BURST combination and BURST and 1 patient (12.5%) used TONIC and BURST. In the SCStol group (*n* = 8), 6 patients (75%) used only one program while 2 patients (25%) used 2 programs. Six patients (75%) used only the TONIC + BURST combination therapy. Among the 2 patients who preferred to have 2 programs in their stimulation portfolio, 1 patient (12.5%) used TONIC stimulation and HF stimulation and 1 patient (12.5%) used TONIC stimulation and BURST stimulation.

At the 6-month follow-up, in the LoC group (*n* = 8), 6 patients used only one program while 5 patients preferred to have 2 programs. Three patients (37.5%) used only TONIC + BURST combination therapy, 2 patients (25%) used TONIC stimulation only (below the paresthesia threshold for 1 patient) and 1 patient (12.5%) used TONIC + HF combination therapy. Among the 2 patients who used 2 programs, 1 patient (12.5%) used TONIC + BURST combination and TONIC stimulation and 1 patient (12.5%) used TONIC and BURST stimulation. In the SCStol group (*n* = 10), 8 patients (80%) used only one program while 2 patients (20%) used 2 programs. Four patients (40%) used only the TONIC + BURST combination therapy, 2 patients (20%) used only BURST stimulation, 1 patient (10%) used only BURST + HF combination therapy and 1 patient (10%) used only TONIC stimulation. Among the 2 patients who preferred to have 2 programs in their stimulation portfolio, 1 patient (10%) used TONIC stimulation and HF stimulation and 1 patient (10%) used TONIC stimulation and BURST stimulation.

At the 12-month follow-up, in the LoC group (*n* = 8), 5 patients (62.5%) used only one program while 3 patients (37.5%) preferred to have 2 programs in their stimulation portfolio. Three patients (37.5%) used only TONIC + BURST combination therapy. TONIC stimulation only was used by 2 patients (25%) (under paresthesia threshold for 1 patient). Among the 3 patients who used 2 programs, 1 patient (12.5%) used TONIC + BURST combination and TONIC, 1 patient (12.5%) used TONIC + BURST combination and BURST and 1 patient (12.5%) used TONIC and BURST. In the SCStol group (*n* = 8), 6 patients (75%) used only one program while 2 patients (25%) used 2 programs. Four patients (50%) used only the TONIC + BURST combination therapy and 2 patients (25%) used only BURST stimulation. Among the 2 patients who preferred to have 2 programs in their stimulation portfolio, 1 patient (12.5%) used TONIC stimulation and HF stimulation and 1 patient (12.5%) used TONIC stimulation and BURST stimulation.

## 4. Discussion

### 4.1. Clinical Potential Added Value of Adapters in Case of FSCSS

While SCS is effective to manage pain in about 50% [1], one of the more challenging conditions is to find solutions in the case of SCS pain relief failure [8,30] or LoE over time [10,31,32]. While the ultimate decision in SCS failure could be IPG and lead(s) explantation [8,11,30], an alternative could nowadays be proposed to the patient with introduction of a device adapter to switch from conventional to independent source spatial targeting [33] and from tonic to new paresthesia-free waveforms, such as burst or HF [19,34,35,36,37].

With a positive conversion rate of 85.2%, following a new screening trial allowed by an adapter implantation on the existing SCS system of patients experiencing a FSCSS characterized by LoE, this retrospective study highlights the clinically significant results observed with new SCS spatial and temporal modalities delivered through new systems, as a salvage therapy. Indeed, a significant reduction in VAS was observed at all follow-ups and the quality of life and functional capacity improved at the 1-, 3- and 6-month follow-up [38]. Objective mapping data allowed us to dissect, within adapter technology, where the added value would be and for which patient this alternative should be prioritized [22].

We observed that regained SCS efficacy was accompanied by paresthesia coverage recovery up to 63.6% at the 12-month follow-up for the LoC group. In addition, pain surface decreased by 51.3% in the LoC group and 26.6% in the SCS tolerance group at the 12-month follow-up. In light of these results, it appears possible to convert a FSCSS back to a new SCS success.

Surprisingly, patient preferences indicated that, despite substantial use of waveform combinations, tonic stimulation was finally adopted by most of our patients at the 12-month follow-up (87.5%, combined with new modalities = 75% or as a standalone waveform = 12.5%). Although Karri et al. [39] showed the superiority of burst over tonic in reduction of pain through a meta-analysis of five studies comparing burst versus tonic, our study demonstrates that patients seem to still appreciate paresthesia-based stimulation. Giving an emphasis to waveforms, Hunter et al. [19] reported significant pain decrease following replacement of the IPG with or without replacement of the lead, in a cohort of 307 patients already implanted with conventional SCS, by using burst as either a salvage therapy or as an upgrade to conventional SCS. Interestingly, they did not show any difference in pain relief between patients who had their entire system changed (leads and IPG) compared with those who simply changed the IPG. This first step suggested the potential use of adapters in patients presenting LoE, while preserving the preexisting leads already in place. Similarly, Andrade et al. [18] showed that combination of multiple waveforms burst and HF, used as a salvage therapy, was safe and efficient to decrease pain in 37 patients already implanted with conventional SCS.

In this study we have reinforced findings previously published, with an added value of our ability to stratify FSCSS patients and to have performed subgroup analysis, allowed by 3D-computerized mapping data and objective metrics. The perspective of using 3D-computerized pain mapping assessment as part of the daily routine could make it possible to better understand the relationships between pain coverage and pain relief, thereby justifying two different electrical approaches, the objective being to modulate either spatial or temporal parameters, in two different patient populations (i.e., loss of coverage and SCS tolerance). The main advantage of this approach resides in its versatility and flexibility. The possibility of combining several innovations in spatial and temporal adjustments, centralized by one technology, should be viewed as an opportunity to solve several patient concerns almost simultaneously. Using an example of one of the patients in this cohort, who was implanted with an adapter, the ability to provide (i) coverage recapturing, (ii) coverage extension, (iii) new pain onset coverage, (iv) comfort perception increase and (v) avoidance of positioning SCS effect, makes it quite easy to understand that the balance of risks/benefits between SCS lead revision, IPG systematic replacement and undergoing a new SCS screening trial thanks to an adapter, connected under local anesthesia, could be in favor when considering this approach, as a salvage therapy, when facing FSCSS. This was clearly documented using objective metrics in this study.

All in all, these results need to be confirmed on a larger controlled prospective cohort with long-term follow-up [37].

### 4.2. Two Patient Profiles to Consider According to FSCSS Pathophysiology, Two Directions to Re-Explore Neural Retargeting but One Philosophy to Delineate, Aiming to Salvage SCS Failure

When patients are experiencing SCS LoE over time, several options can be discussed in a multidisciplinary pain reassessment, such as lead revision [24] or Internal Pulse Generator (IPG) upgrade [18,19,24,37,39,40]. Thanks to recent advances in SCS hardware and programming technologies, patients can have access to various waveforms (standard rate, high frequency (HF), burst, high dose (HD)) [16,33,37,41,42] and tailored advanced targeting (multisource IPG) [17,18] of the chronic pain experienced by SCS-implanted patients [43,44].

As described in the introduction, FSCSS patients might observe LoE with or without LoC. This also largely depends on the type of SCS program(s) used: tonic stimulation and/or sub-paresthetic stimulation modalities. Some authors state that LoE is preferentially observed with patients using tonic stimulation [39]. It is interesting to note that in our cohort all FSCSS patients, independently of their initial device, had the opportunity to try waveforms other than tonic stimulation, HD and Differential Target Multiplexed by Medtronic, BurstDR by Abbott and burst and HF by Boston Scientific. Therefore, it is not possible to conclude that SCS failure, due to LoE, results from a non-availability of sub-perception waveforms, and we must admit that their potential use, aiming to solve the problem, left these patients in a failed SCS condition.

Moreover, independently of the waveform chosen and used by the patients, the majority of these FSCSS patients, considered for this study, were complaining of LoE, without LoC, at their initial evaluation:

− The first group includes patients facing LoC (*n* = 11), which potentially leads to LoE, and, surprisingly, once again, independently of the waveform used. This lack/loss of coverage might also result from neural plasticity, but the fact that some neurons were adequately depolarized to create an analgesic effect initially, but not longer, could correspond to the notion of “SCS resistance” rather than “SCS tolerance”. There appears to be a physical, architectural change in the localization of the targeted fibers (the so-called “sweet spot”), and the fact that we were able to spatially recapture all patient sweet spots with adapters, and that they remained stable within time, shows that: (i) this is a spatial problem, and that (ii) it was probably a technological limitation in the method of delivering the electrical field, which was responsible for previous SCS device failure to recapture the new target. This spatial recapturing corresponds to a major added value of the technology connected to the adapter. Arising from this argumentation, we can conclude that LoE recovery, through LoC spatial recapturing, is clearly not to be attributed to any waveform change or temporal resolution adjustment of the electrical signal. We can thereby assume that “SCS resistance”, characterizing this first group of patients, is a topographic matter. This topographic matter has been addressed by spatial optimization of the signal in this study.
− Examining the specificities of this new technology, the difference in terms of spatial targeting abilities, compared to other SCS systems, might come from anatomically guided neural targeting, using a three-dimensional finite element model of the spinal cord, derived from the Twente University computational bio-electrical model [45,46]. This model is applied to shape the electrical field, when delivering paresthesia-based SCS therapy, through multi-source generation of the electrical field, involving independent electrical sources at the level of the IPG. This signal processing, called “Multiple Independent Current Control (MICC^TM^)”, places a dynamic but constant electrical field along dorsal horn intra-spinal structures, in axial distribution, as well as a uniform electrical influence on longitudinal spinal cord tracts, thereby optimizing “sweet spot” targeting.
− The second group includes patients facing LoE, without LoC, who have developed the notion of “SCS tolerance” (*n* = 16), leading to another type of FSCSS. The notion of electric tolerance has been introduced more recently and is still the subject of debate. Tolerance could correspond either to local neurochemical adaptation to the electrical field delivered by the SCS lead [47] or to neurochemical “desensitization” at the level of spinal pathways/supra-spinal projections, even at the cortical integration level [48,49] or local architectural plasticity, including fibrotic developments, or else neural plasticity observed at the level of the central nervous system pathways/cortex. It is interesting to note that SCS tolerance has been reported in both sub-categories of stimulation: tonic stimulation and novel sub-perception waveforms [50,51]. The reasons for this phenomenon are not completely understood, but neural plasticity and fibrosis around the electrodes have been postulated as contributing factors [13]. When exposed to the same stimulation input in the long term, changes in synaptic connections may indeed occur in the pain transmission neural network, which becomes less sensitive to SCS. This neural adaptation could explain why some patients lose the therapeutic effect over the long term despite a stable electrical field producing adequate pain coverage.
− It appears here, in contrast to the LoC group, that the solution might not come from spatial retargeting, since the electric field generated by the new SCS device stimulates the same fibers as the initial device, while the susceptibility of these fibers has changed and could benefit from non-spatial modalities. This has been achieved in this study by temporal adjustments of the electrical signal, with a clinically significant outcome, for this specific population, using various waveforms and combinations (such as tonic in combination with burst or HF). As an echo to recent data regarding the interest of combination therapy and the ability to customize programs for patients so as to give them a certain level of autonomy to combine waveforms [16,52] by cycling them when necessary (e.g., patients switching to sub-perception mode when sleeping), our findings should help to personalize the therapy, encompassing patient variability in terms of electrical susceptibility, and even tolerance.

### 4.3. Proposal of a Salvage SCS Algorithm for Failed SCS Syndrome (FSCSS)

**Figure 5:**
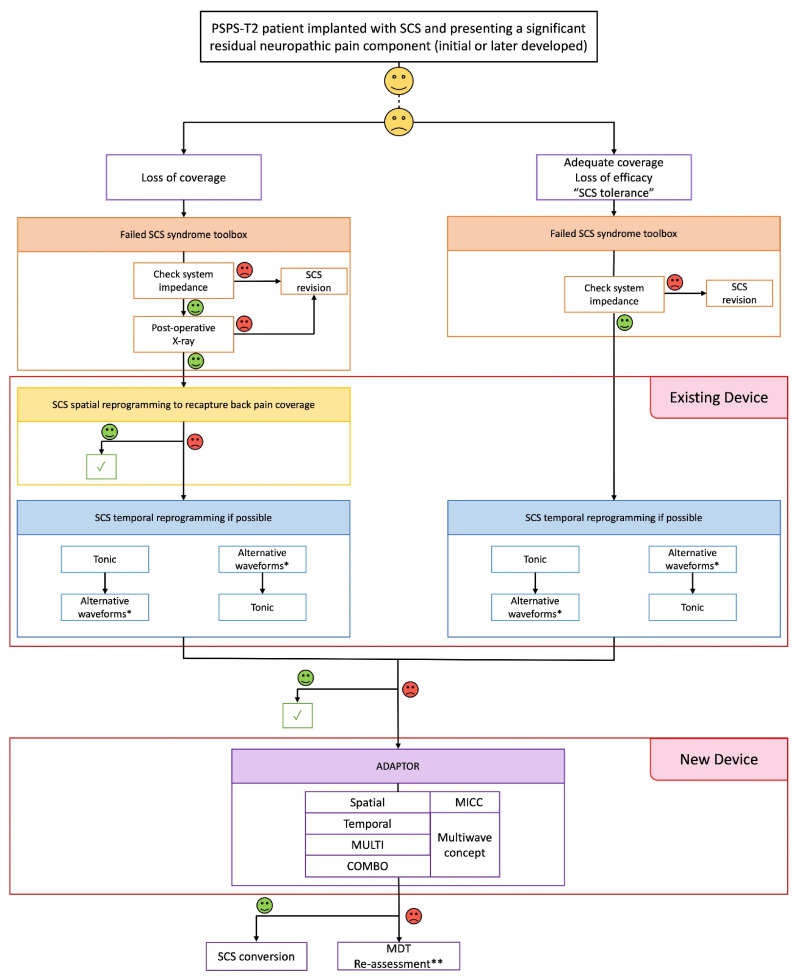
A theoretical proposal of SCS rescue algorithm in case of failed SCS syndrome.

In light of these study results, the optimization of the use of adapters, the target population and their place in our therapeutic armamentarium can be discussed. Our ambition is to describe general principles of a theoretical salvage algorithm (Figure 5), proposing to implant an adapter and to test the capacity of alternative technologies to perform spatial and/or temporal retargeting, if it has been proven impossible to obtain it by reprogramming the existing SCS device. This theoretical algorithm should be validated on a larger cohort and can be used as a baseline for designing future clinical studies and to try to better address PSPS-T2 patients experiencing FSCSS. The following proposals are made based on the distinction between our two subgroups of FSCSS patients.

#### 4.3.1. Proposal for LoC Group: SCS Resistance Characterized by LoE Associated with LoC

Facing a chronic pain patient, already implanted with SCS, with a loss of efficacy due to loss of coverage, (and as a result, under tonic stimulation), after checking for impedance dysfunction and for lead potential displacement (with X-ray), we can propose to convert to an adapter and to use a spatial approach with MICC first, aiming to recapture the sweet spot, if possible [33], and to switch, as a second step, to a temporal approach, by proposing one or several available waveform(s) (presented by * in Figure 5), comprising burst, HF, a combination of them, or a combination with tonic stimulation, which can be delivered synchronously, or cycled, based on patient preference [16].

#### 4.3.2. Proposal for SCStol Group: SCS Tolerance Characterized by LoE without LoC

Facing a chronic pain patient, already implanted with SCS, with an adequate coverage but insufficient or lack of pain relief of their neuropathic pain component, our approach consists of: (i) checking impedance systematically (adequate coverage presumes that, at least at the level of involved contacts, impendences are acceptable) and checking hardware dysfunction (idem: no particular reason here since paresthesia cover the appropriate painful territory) and (ii) proposing conversion to a new screening trial, through adapters, allowing for temporal retargeting with a different technology, following the same modalities as those presented above, and if the trial is positive, SCS IPG is replaced without any change in the lead(s).

We propose to reassess patients’ refractory to all available measures presented in this salvage algorithm (** in Figure 5), in a Multidisciplinary Team (MDT) context (i) to reconsider patient selection, (ii) to rule out any etiology that would require a different approach than neurostimulation, including re-imaging, clinical re-evaluation and a new psychological assessment.

In case the indication of neurostimulation is still relevant, with positive predictive factors regarding patient selection, with no clear indication to any further surgery or invasive procedure to treat the potential pain generator and in the event that the conclusion of MDT assessment is oriented towards an SCS technical failure, which is not accessible to revision, one last option would be to discuss changing the anatomical target of neurostimulation (DRG stimulation, PNfS, etc.), given the fact that 4 ports, instead of 2, are now available in this new generation of IPGs, making it possible to manage 4 different leads, with 32 contacts.

### 4.4. Study Limitations

Despite its originality, this study suffers from several limitations. First, our small sample size should be considered as nuancing the power of our results and consequently our interpretation. In addition, available waveform profiles were limited. Our limited sample size did not offer us the opportunity to have many patients/waveform groups and the relatively high number of available choices, especially when combination therapy is applied, cannot reflect all possibilities of stimulation modalities. However, interestingly, most patients went back to tonic, with or without a combination therapy. This must be confirmed on larger sample size studies. In addition, the retrospective nature of the study implies consideration of our promising results as a starting point for future prospective studies. The observational nature of the study led to an inability to control for unobserved confounders, a point which could be improved in future controlled randomized study designs.

In case of FSCSS, with a patient implanted with hybrid stimulation (e.g., SCS + DRG, SCS + PNfS, etc.), we could wonder if the potential new IPG, implanted following a positive lead trial, would not interfere with the other neurostimulation targets. Ongoing studies [53] and recent publications [24] consider this important question. We should note that one of the advantages of this new technology is to propose four channels of stimulation, with a multi-source generation of the current. This could open the gate to multi-target hybrid therapies, in case of potential developments.

## 5. Conclusions

This study highlights the clear added value of implanting adapters to test alternative SCS modalities, when a patient is experiencing SCS failure despite good initial results, after checking hardware dysfunction and extensive SCS reprogramming with the existing implanted device. Whether FSCSS is a consequence of SCS resistance or of SCS tolerance, a salvage algorithm could guide patients and implanters, through a standardized reprogramming approach, towards a possible conversion from FSCSS back to an SCS success. Objective quantitative pain and paresthesia 3D mapping assessments appear to be of importance, in order to (i) more precisely document the potential cause of FSCSS, which might correspond to a specific patient profile, and (ii) to delineate objective comparisons between devices and targets, helping us to hierarchize our therapeutic options in the near future, by coupling these metrics into multidimensional composite pain indexes.

## 6. Patents

Patent application N° PCT/EP2014/067231

Patent application N° PCT/FR 14/000 186

Patent application N° PCT/FR 14/000 187

## Figures and Tables

**Table 1:** Patients baseline sociodemographic and clinical characteristics.

Variable	(*n* = 27)
Age mean ± SD	53.2 ± 11.6
**Sex**: Female/Male (*n*, percentage)	12 (44.4%)/15 (55.6%)
**Lead type**	
Octade	45 (18.5%)
Quad	4 (14.8%)
Octrode	1 (3.7%)
Penta	3 (11.1%)
Octade + quad	1 (3.7%)
5-6-5	3 (11.1%)
Octade + linear 3-6	2 (7.4%)
Vectris	2 (7.4%)
5-6-5 + linear 3-6	12 (37.84%)
5-6-5 + octade	1 (3.7%)
5-6-5 + quad	1 (3.7%)
vectris + octade	1 (3.7%)
Infinion + octade	1 (3.7%)
Octade	45 (18.5%)
Quad	4 (14.8%)
**Implantation**	
Percutaneous	7 (26.9%)
Subcutaneous	2 (7.4%)
Surgical	9 (33.3%)
Percutaneous + subcutaneous	5 (18.5%)
Surgical + subcutaneous	4 (14.8%)
**Pain localization**	
Leg pain	2 (7.4%)
Upper limbs pain	3 (11.1%)
Cluster headache	1 (3.7%)
Back and leg pain	19 (70.4%)
Groin pain	2 (7.4%)
**Lead placement**	
Thoracic	13 (48.1%)
Cervical	2 (7.4%)
Occipital	1 (3.7%)
Thoracic + subcutaneous	9 (33.3%)
Conus terminalis	2 (7.4%)
Duration between previous SCS and adapter implantation (years)	5.9 (5.2)
**Follow-up duration**	
Baseline	27
1 month	19/27 (70.4%)
3 months	16/27 (59.3%)
6 months	18/27 (66.7%)
12 months	16/27 (59.3%)
Baseline VAS (mean ± SD)	75.1 (14.9)
Baseline ODI (mean ± SD)	48.5 (15.9)
Baseline EQ5D (mean ± SD)	0.25 (0.2)

SCS: Spinal Cord Stimulation; VAS: Visual Analogic Scale; ODI: Oswestry Disability Index; EQ-5D-5L: EuroQol-5 Dimensions 5 Levels.

**Table 2:** Outcomes comparisons between baseline and 1-month, 3-month, 6-month and 12-month follow-ups (*n* = 19).

Outcomes	Before Mean ± SD	After Mean ± SD	CI95% of the Difference	*p*-Value
1-month follow-up (*n* = 19)				
Global VAS	75.26 ± 16.45	45.56 ± 24.31	[16.04; 43.96]	0.001
ODI score	48.81 ± 12.55	31.69 ± 12.78	[6.61; 23.21]	0.001
EQ-5D index	0.25 ± 0.17	0.53 ± 0.23	[−0.38; −0.13]	0.004
HADS depression	5.82 ± 3.52	3.6 ± 2.56	[0; 4]	0.044
HADS anxiety	8.88 ± 4.55	6.53 ± 3.78	[0; 4]	0.049
Pain surface (cm²)	938.26 ± 840.56	441.82 ± 597.72	[182.96; 903.51]	0.001
Very intense pain surface (cm²)	638.33 ± 862.57	28.24 ± 82.65	[192; 1103.3]	0.002
Perceived pain relief		68.3/100 ± 21.6		
PGIC		6.1 ± 1.0		
3-month follow-up (*n* = 16)				
Global VAS	71.88 ± 16.82	46.25 ± 19.96	[13.17; 38.08]	0.005
ODI score	45.4 ± 16.97	28.12 ± 12.36	[5.22; 36.23]	0.020
EQ-5D index	0.29 ± 0.21	0.53 ± 0.19	[−0.45; −0.07]	0.021
HADS depression	6.08 ± 3.87	3.5 ± 3.03	[0.26; 6.41]	0.042
HADS anxiety	8.0 ± 4.84	6.7 ± 3.8	[0.08; 3.25]	0.056
Pain surface (cm²)	919.75 ± 926.34	569.79 ± 878.56	[68.09; 403.77]	0.014
Very intense pain surface (cm²)	493.12 ± 920.89	288.5 ± 913.49	[−57.82; 507.82]	0.021
Perceived relief		72.2 ± 14.8		
PGIC		6.0 ± 0.7		
6-month follow-up (*n* = 18)				
Global VAS	78.33 ± 12	36.67 ± 23.26	[28.76; 54.57]	<0.001
ODI score	43.11 ± 15.77	31.9 ± 13.85	[−0.12; 25.52]	0.042
EQ-5D index	0.32 ± 0.22	0.53 ± 0.26	[−0.43; −0.08]	0.022
HADS depression	5.07 ± 3.34	4.3 ± 3.89	[0.61; 2.51]	0.019
HADS anxiety	7.93 ± 4.62	6.6 ± 6.15	[−0.1; 2.54]	0.055
Pain surface (cm²)	848.22 ± 778.08	711.89 ± 969.23	[−106.8; 379.47]	0.088
Very intense pain surface (cm²)	562.53 ± 903.3	223.61 ± 809.48	[45.48; 606.05]	0.021
Perceived relief		72.8 ± 21.9		
PGIC		5.8 ± 0.8		
12-month follow-up (*n* = 16)				
Global VAS	78.12 ± 11.09	33.75 ± 21.87	[33.03; 55.72]	<0.001
ODI score	47.71 ± 14.62	34 ± 22.84	[−6.66; 73.86]	0.098
EQ-5D index	0.27 ± 0.19	0.48 ± 0.36	[−0.88; 0.1]	0.181
HADS depression	6.33 ± 3.85	5 ± 5.55	[−2.13; 13.13]	0.125
HADS anxiety	8.75 ± 5.19	8 ± 5.8	[−1.5; 8]	0.181
Pain surface (cm²)	887 ± 804.4	545.12 ± 859.58	[60.19; 623.56]	0.012
Very intense pain surface (cm²)	573.4 ± 950.46	240.12 ± 858.75	[16.84; 617.7]	0.019
Perceived relief		67.5 ± 28.9		
PGIC		6.4 ± 0.5		

VAS: Visual Analogic Scale; ODI: Oswestry Disability Index; EQ-5D: EuroQoL 5-Dimensions; HADS: Hospital Anxiety and Depression Scale; PGIC: Patient Global Impression of Change.

**Table 3:** Outcome comparisons between baseline and last follow-up visit (*n* = 27).

Outcomes	Before Mean ± SD	After Mean ± SD	CI95% of the Difference	*p*-Value
Global VAS	75.06 ± 14.91	39.13 ± 27.90	[23.7; 48.16]	<0.001
ODI score	48.48 ± 15.93	38.89 ± 20.45	[2.05; 28.15]	0.021
EQ5D	0.25 ± 0.20	0.39 ± 0.30	[−0.34; −0.05]	0.023
HADS depression	6.19 ± 3.71	5.08 ± 4.55	[−0.45; 5.25]	0.067
HADS anxiety	8.52 ± 4.32	7.50 ± 4.15	[0.35; 3.65]	0.036
Pain surface	1085.19 ± 1360.69	718.85 ± 1430.0	[117.26; 509.82]	0.001

VAS: Visual Analogic Scale; ODI: Oswestry Disability Index; EQ-5D: EuroQoL 5-Dimensions; HADS: Hospital Anxiety and Depression Scale.

**Table 4:** Comparisons of percentage of pain intensity decrease, percentage of pain surface decrease and paresthesia coverage between the “loss of coverage” group (*n* = 11) and the “SCS tolerance” group (*n* = 16).

Variable	LoC Group (*n* = 11)	SCStol Group (*n* = 16)	*p*-Value
Age	54.6 ± 11.6	52.2 ± 11.8	0.68
Sex			0.76
Male	7 (63.6%)	8 (50.0%)	
Female	4 (36.4%)	8 (50.0%)	
Duration between SCS implantation and adapter rescue therapy (years)	6.4 ± 5.1	5.6 ± 5.4	0.73
1-month follow-up	*n* = 10	*n* = 9	
Percentage of VAS decrease	32.56 ± 41.81	26.13 ± 33.33	0.60
Percentage of pain surface decrease	64.3 ± 33.86	11.77 ± 65.81	0.034
Paresthesia coverage	73.88 ± 35.77	27.6 ± 32.99	0.048
3-month follow-up	*n* = 8	*n* = 8	
Percentage of VAS decrease	36.89 ± 29.98	31.75 ± 39.61	0.75
Percentage of pain surface decrease	47.01 ± 38.69	20.13 ± 69.85	0.047
Paresthesia coverage	55.57 ± 46.23	30.5 ± 47.49	0.35
6-month follow-up	*n* = 8	*n* = 10	
Percentage of VAS decrease	50.56 ± 31.83	36.36 ± 43.69	0.59
Percentage of pain surface decrease	34.47 ± 44.77	18.13 ± 64.32	0.60
Paresthesia coverage	51.25 ± 48.66	38.56 ± 52.7	0.92
12-month follow-up	*n* = 8	*n* = 8	
Percentage of VAS decrease	58.68 ± 32.79	35.77 ± 44.09	0.19
Percentage of pain surface decrease	51.32 ± 42.31	26.61 ± 54.39	0.21
Paresthesia coverage	63.62 ± 46.26	21.5 ± 47.25	0.14

SCS: Spinal Cord Stimulation; VAS: Visual Analogic Scale.

## Data Availability

Not applicable.
